# New paradigm for stage III melanoma: from surgery to adjuvant treatment

**DOI:** 10.1186/s12967-019-2012-2

**Published:** 2019-08-14

**Authors:** Paolo Antonio Ascierto, Lorenzo Borgognoni, Gerardo Botti, Michele Guida, Paolo Marchetti, Simone Mocellin, Paolo Muto, Giuseppe Palmieri, Roberto Patuzzo, Pietro Quaglino, Ignazio Stanganelli

**Affiliations:** 1 Unit Melanoma, Cancer Immunotherapy and Innovative Therapies, Istituto Nazionale Tumori IRCCS Fondazione “G. Pascale”, Naples, Italy; 2 Ospedale Santa Maria Annunziata and University of Florence, Florence, Italy; 3 Istituto Nazionale Tumori IRCCS Fondazione “G. Pascale”, Naples, Italy; 4 Unit Melanoma and Rare Tumors, IRCCS Istituto Tumori Giovanni Paolo II, Bari, Italy; 5 Oncologia Medica B Policlinico Umberto I di Roma, Rome, Italy; 6 Surgical Oncology Unit, IOV-IRCCS of Padova and Dept. Surgery Oncology Gastroenterology, University of Padova, Padua, Italy; 7 Unit of Cancer Genetics, ICB-CNR, Sassari, Italy; 8 Research Director CNR, Italian Melanoma Intergroup (IMI), Unit of Cancer Genetics, Head Institute of Biomolecular Chemistry (ICB), National Research Council (CNR), Sassari, Italy; 9 IRCCS Fondazione Istituto Nazionale dei Tumori di Milano, Milan, Italy; 10 Dermatologic Clinic, Department of Medical Sciences, University of Turin Medical School, Turin, Italy; 11 Skin Cancer Unit, Istituto Scientifico Romagnolo per lo Studio e la Cura dei Tumori (IRST), IRCCS, Meldola, FC Italy; 12 University of Parma, Parma, Italy

**Keywords:** Staging, Surgery, Adjuvant treatment, Melanoma, Lymph node dissection

## Abstract

**Background:**

Recently the 8th version of the American Joint Committee on Cancer (AJCC) classification has been introduced, and has attempted to define a more accurate and precise definition of prognosis in line with the major progresses in understanding the biology and pathogenesis of melanoma. This new staging system introduces major changes in the stage III staging system. Indeed, surgical practice is changing in stage III patients, since, according to recent evidence, there is no survival benefit in radical lymph node dissection following a positive sentinel lymph node dissection. Therefore, some patients currently staged IIIB-C after dissection could be downgraded to IIIA (as in the case of patients with metastatic non-sentinel lymph nodes) since many completion lymph node dissections will no longer be performed. Moreover, new and effective targeted and immune strategies are being introduced in the pharmacological armamentarium in the adjuvant setting, showing major efficacy.

**Conclusions:**

This article provides the authors’ personal view on the above-mentioned topics.

## Background

The American Joint Committee on Cancer (AJCC) classification has represented the reference staging system for melanoma for many decades. Recently, the AJCC 8th version has been introduced, and it has attempted to define a more accurate and precise definition of prognosis in line with the major progresses in understanding the biology and pathogenesis of melanoma [1].

However, this new staging system has been largely criticized [2]. For instance, it uses the same histological factors of the AJCC 7th version, and although with some refinements, it does not introduce any new prognostic biomarkers; moreover, in stages I–III melanoma, the AJCC 8th version is still based on 5- and 10-year melanoma-specific survival, and does not take into account the increased survival in stage IV melanoma associated with the introduction of new therapies. Even more importantly, the AJCC 8th version introduces major changes in the stage III staging system [2]. Indeed, surgical practice is changing in stage III patients, since two studies have shown that there is no survival benefit in radical lymph node dissection after a positive sentinel lymph node dissection [3, 4]. Therefore, some patients currently staged IIIB–C after dissection could be downgraded to IIIA (as in the case of patients with metastatic non-sentinel lymph nodes) since many completion lymph node dissections (CLND) will no longer be performed. Moreover, new and effective targeted and immune strategies are being introduced in the pharmacological armamentarium in the adjuvant setting, showing major efficacy [5–7]. Other trials are ongoing (Table 1), and others will start, likely leading to complete changes in the therapeutic approach for stage III melanoma.

**Table 1:** Ongoing major trials in stage III melanoma

Trial name	Trial ID	Aim
Checkmate 915	NCT03068455	To determine whether nivolumab + ipilimumab, is more effective than nivolumab alone, in delaying recurrence in patients with complete surgical removal of stage IIIb/c/d or stage IV melanoma
ECOG 1619	NCT01274338	To compare adjuvant ipilimumab with high-dose interferon alfa-2b in treating patients with high-risk stage III–IV melanoma that has been removed by surgery
SWOG S1414 [27]	MCT02506153	Randomized trial comparing standard of care to pembrolizumab in patients at high risk for recurrence and death after surgery

This article provides the authors’ personal view on the above-mentioned topics.

## Complete dissection or observation for patients with sentinel node metastasis

**Fig. 1:**
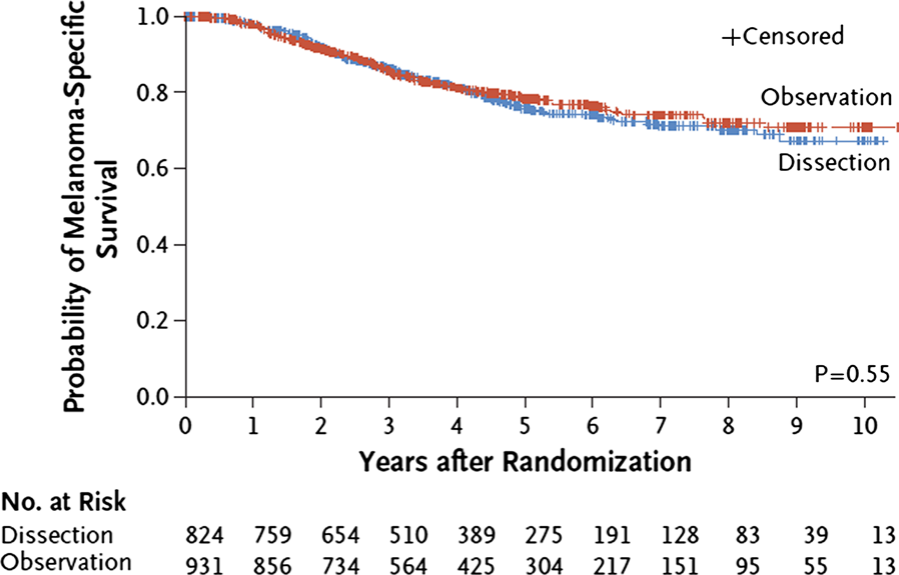
Melanoma-specific survival following completion lymph-node dissection or observation in the per-protocol analysis of the trial by Faries et al. [4]. Reproduced with permission

In a recent landmark international trial, Faries et al. randomly assigned patients with sentinel node metastases to lymph node dissection (dissection group; n = 824) or nodal observation with ultrasonography (observation group; n = 931) [4]. Patients in the dissection group did not show improved melanoma-specific survival compared with those in the observation group (86 ± 1.3% vs 86 ± 1.2%) at a median follow-up of 43 months (Fig. 1) [4]. However, the rate of disease-free survival was slightly higher in the dissection group (68 ± 1.7% vs 63 ± 1.7%; p = 0.05), given an increased rate of disease control in the regional nodes at 3 years (92 ± 1.0% vs 77 ± 1.5%; p < 0.001). Statistical analysis revealed that non-sentinel node metastases (reported in 11.5% of the patients in the dissection group) represent an independent prognostic factor for recurrence (hazard ratio [HR]: 1.78; p = 0.005). Overall, these findings demonstrate that immediate completion of lymph node dissection can increase the rate of regional disease control and provide prognostic information, without actually improving melanoma-specific survival. However, the patients in this study were a highly selected group with an extremely low burden of sentinel node disease (median tumor burden < 1 mm and only one node involved in 70% of patients). Similar results were reported in another pivotal multicenter, phase III trial by Leiter et al. [3]. In this latter study, which was terminated early due to difficulties in enrollment and low event rate, patients were randomly assigned to either CLND (n = 243) or observation (n = 233) [3]. Distant metastasis-free survival at 3 years was 74.9% (90% CI 69.5–80.3) following CLND and 77.0% (90% CI 71.9–82.1) in the observation group, similarly no differences in overall survival (OS) were found between the two groups.

According to the above-mentioned bases, there appears to be no survival benefit associated with CLND, although this strategy can lead to more refined staging and regional node control. For instance, a recent retrospective study from the European Organisation for Research and Treatment of Cancer (EORTC), including 1015 patients, showed that CLND led to upstaging in N-category in 19% and in AJCC stage in 5–6% [8]. Similar findings were reported by Madu et al. [9].

Active surveillance for patients who do not undergo CLND following a positive sentinel lymph node should include nodal ultrasound as a component of the follow-up strategy. CLND should be discussed with patients clarifying the risks, benefits and alternatives of the procedure, including their overall risk of harboring metastatic non-sentinel lymph nodes and the impact of dissection on staging, regional control and survival. In this regard, the Italian Melanoma Intergroup has developed a nomogram to predict the risk of non-sentinel node positivity, which can help clinicians to discuss with patients the opportunity of CLND [10].

However, with the advent of the new adjuvant treatments, there is probably less need for indicating a CLND.

## The importance of adjuvant therapy

In light of the evolving landscape of adjuvant therapy in melanoma and the lack of survival benefit of CLND, it becomes important to explore possible consequences of omitting CLND, and whether it is possible to stratify positive sentinel node patients on the basis of information retrieved from sentinel lymph node biopsy [8]. Verver et al. in a retrospective analysis from nine EORTC melanoma group centers, showed that adequate stratification of patients with sentinel lymph nodes was possible based on ulceration and tumor burden category, with 1 mm being the threshold to distinguish between low-/intermediate- and high-risk patients [8]. In particular, the identification of low-, intermediate- and high-risk patients could help select adjuvant therapy in clinical practice.

To this end, the therapeutic efficacy of adjuvant treatment with IFN-α for the treatment of patients with AJCC stage II–III cutaneous melanoma in terms of both disease-free survival and, to a lower extent, OS was already shown in two pivotal meta-analyses [11, 12]. More recently, a number of studies have investigated adjuvant therapy with newly introduced treatments, and therefore since 2011 the treatment of advanced or metastatic melanoma has undergone a sort of revolution with the introduction, which has also been recently evaluated in an adjuvant setting in a number of randomized clinical trials (Table 2) [13].

**Table 2:** Summary of clinical results reported in the main adjuvant trials with new drugs

Author	Study	Recruitment years	No. patients	Inclusion	Treatment	HR for PFS	95% CI	2-year PFS	3-year PFS	4-year PFS	5-year PFS
Eggermont et al. [16]	EORTC 18071	2008–2011	951	III (> 1 mm)	Ipilimumab vs placebo	0.75	0.64–0.90		46.5%		40.8%
Weber et al. [5]	CheckMate238	Mar–Nov 2015	906	IIIB-IIIC/IV	Nivolumab vs ipilimumab	0.65	0.51–0.83	63%	–	–	–
Long et al. [6]	COMBI-AD	2013–2014	870	III (> 1 mm)	Dabrabenif/trametinib vs placebo	0.49	0.40–0.59	67%	59%	54%	
Maio et al. [14]	BRIM8	2012–2015	498	IIC, IIIA, IIIB IIIC	Vemurafenib vs placebo	0.54 0.80	0.37–0.78 0.54–1.18	72.3% 46.3%			
Eggermont et al. [7]	KeyNote 054	2015–2016	1019	III (> 1 mm)	Pembrolizumab vs placebo	0.57	0.43–0.74	71.4% (18 months)	–	–	–

### Vemurafenib

The phase III, international, double-blind, randomized, placebo-controlled BRIM8 trial evaluated adjuvant vemurafenib monotherapy in melanoma [14]. In total, 498 patients with histologically confirmed stage IIC–IIIA–IIIB or stage IIIC *BRAF*^V600^ mutation-positive melanoma that had been fully resected were randomly assigned to either twice-daily adjuvant oral vemurafenib or placebo for 52 weeks. The primary endpoint was disease-free survival, which was evaluated separately in each cohort. Median follow-up was 33.5 months in patients with stage IIIC and 30.8 months in those with stage IIC–IIIA–IIIB disease. In the former cohort, median disease-free survival was 23.1 months (95% CI 18.6–26.5) with vemurafenib and 15.4 months (95% CI 11.1–35.9) with placebo (HR: 0.80, 95% CI 0.54–1.18; p = 0.26). In the latter cohort (patients with stage IIC–IIIA–IIIB disease), median disease-free survival was not reached in the vemurafenib group compared with 36.9 months (95% CI 21.4–not estimable) in the placebo group (HR: 0.54; 95% CI 0.37–0.78; p = 0.0010); however, statistical significance was not reached because of the prespecified hierarchical prerequisite for the primary disease-free survival analysis. Moreover, the particular trend of the disease-free survival curve in the vemurafenib group, which shows an increase in the incidence of relapse after some time on treatment, does suggest that BRAF inhibitors alone may not be sufficient to prevent relapse. These findings suggest that adjuvant vemurafenib may not be considered an optimal treatment regimen in this patient population.

### Dabrafenib + trametinib

In the randomized COMBI-AD phase III trial, patients with resected *BRAF*^V600^-mutant stage III melanoma (IIIA with deposits more than 1 mm, IIIB–IIIC) were assigned to 12 months’ adjuvant dabrafenib + trametinib or placebo [6, 15]. At a median follow-up of 2.8 years, the estimated 3-year rate of relapse-free survival (RFS) was 58% with the combination therapy versus 39% with placebo group (HR: 0.47, 95% CI 0.39–0.58; p < 0.001). The 3-year OS rates were 86% and 77%, respectively (HR: 0.57, 95% CI 0.42–0.79; p = 0.0006), but this level of improvement did not cross the prespecified interim analysis boundary to claim statistical significance (based on a prespecified threshold of p = 0.000019). Rates of distant metastasis-free survival and freedom from relapse were also higher with dabrafenib + trametinib compared with placebo.

At the updated analysis of the COMBI-AD trial, with a median follow-up of 44 (dabrafenib + trametinib) or 42 months (placebo), 3- and 4-year RFS rates were 59% (95% CI 55–64%) and 54% (95% CI 49–59%) with dabrafenib + trametinib, and 40% (95% CI 35–45%) and 38% (95% CI 34–44%) with placebo, respectively (HR: 0.49, 95% CI 0.40–0.59) [15]. Similarly, distant metastasis-free survival was longer with dabrafenib + trametinib (HR: 0.53, 95% CI 0.42–0.67). The estimated cure rate was 54% (95% CI 49–59%) in the dabrafenib + trametinib arm compared with 37% (95% CI 32–42%) in the placebo arm. Subgroup analysis showed that dabrafenib + trametinib benefited patients regardless of different baseline factors, including disease stage, nodal metastatic burden and ulceration.

At the American Society of Clinical Oncology (ASCO) 2018 meeting, the patients included in the COMBI-AD trial were re-staged according to the new AJCC 8th edition system. The benefit of dabrafenib + trametinib was observed across all AJCC 8th edition subgroups in resected high-risk stage III melanoma patients, even if it was less evident and not reaching statistical significance for stage IIIA.

A biomarker analysis of the COMBI-AD trial was recently presented. MAPK pathway gene alterations did not correlate with outcomes, while immune gene-expression signatures (e.g., IFN-γ) were strongly prognostic in both arms. High tumor mutational burden added positive prognostic value to IFN-γ signature in the placebo arm, whereas in the combination arm, it identified patients with longer RFS independently of tumor mutational burden. Remarkably, at this analysis, the rate of loco-regional recurrence (without distant involvement) with dabrafenib + trametinib was 32% compared with 43% with placebo. Rates of distant-only recurrences were 59% and 51%, respectively.

### Ipilimumab

Eggermont et al. conducted a phase III trial to evaluate adjuvant ipilimumab (10 mg/kg) in patients with complete resection of stage III melanoma [16]. Patients were randomly assigned to ipilimumab (n = 475) or placebo (n = 476) for up to 3 years or until disease recurrence or an unacceptable level of toxic effects occurred. At a median follow-up of 5.3 years, the 5-year rate of RFS (primary endpoint) was 40.8% with ipilimumab and 30.3% with placebo group (HR: 0.76, 95% CI 0.64–0.89; p < 0.001). At 5 years, the rates of OS were 65.4% and 54.4%, respectively (HR: 0.72, 95.1% CI 0.58–0.88; p = 0.001), and the rates of distant metastasis-free survival were 48.3% and 38.9%, respectively (HR: 0.76, 95.8% CI 0.64–0.92; p = 0.002). Grade 3–4 adverse events were reported in 54.1% of the patients on ipilimumab group and in 26.2% of those assigned to placebo; grade 3–4 immune-related adverse events occurred in 41.6% of patients (fatal in five cases, 1.1%) on ipilimumab group versus 2.7% of those taking placebo. The authors concluded that ipilimumab may represent an effective adjuvant therapy for high-risk stage III melanoma, although the high rates of immune-related adverse events with ipilimumab can represent a concern.

### Nivolumab

In a randomized, double-blind, phase III trial, Weber et al. evaluated the efficacy of nivolumab compared with ipilimumab, for adjuvant therapy in patients with resected advanced melanoma [5]. In total, 906 patients undergoing complete resection of stage IIIB, IIIC or IV melanoma were assigned to either nivolumab (3 mg/kg every 2 weeks) or ipilimumab (10 mg/kg every 3 weeks for four doses and then every 12 weeks), for a maximum period of 1 year. In the presence of recurrence, patients could cross-over to pembrolizumab if randomized in the placebo arm or repeat pembrolizumab (recurrence more than 6 months after the end of treatment). At a minimum follow-up of 18 months, the 12-month rate of RFS higher with nivolumab than with ipilimumab (70.5% [95% CI 66.1–74.0] vs 60.8% [95% CI 56.0–65.2]; HR: 0.65, 97.56% CI 0.51–0.83; p < 0.001). Grade 3–4 treatment-related adverse events were reported in 14.4% of the patients on nivolumab and 45.9% of those on ipilimumab group, with a lower discontinuation rate for adverse events with nivolumab (9.7% vs 42.6%). These data showed that adjuvant therapy with nivolumab among patients undergoing resection of stage IIIB, IIIC or IV melanoma can result in longer RFS and a lower rate of grade 3–4 adverse events compared with adjuvant ipilimumab.

### Pembrolizumab

Adjuvant therapy with pembrolizumab in patients with resected, high-risk stage III melanoma was evaluated in the recently published phase III, double-blind KEYNOTE-054 trial (EORTC 1325) [7]. Patients were randomly assigned to either pembrolizumab (n = 514) or placebo (n = 505) for a maximum period of approximately 1 year. At a median follow-up of 15 months, patients on pembrolizumab showed a higher 1-year rate of RFS than placebo (75.4% [95% CI 71.3–78.9] vs 61.0% [95% CI 56.5–65.1; HR: 0.57, 98.4% CI 0.43–0.74; p < 0.001). This finding was consistent in the 853 patients with PD-L1-positive tumors (77.1% vs 62.6%; HR: 0.54, 95% CI 0.42–0.69; p < 0.001). Grade 3–5 treatment-related adverse events were reported in 14.7% of patients in the pembrolizumab group and in 3.4% of patients in the placebo group. One treatment-related death (myositis) occurred in the pembrolizumab group.

At the Society for Melanoma Research (SMR) 2018 meeting, the prognostic and predictive value of AJCC-8 staging in the KEYNOTE-054 trial of pembrolizumab were presented [17]. Remarkably, the application of the AJCC-8 classification allowed to identify subgroups with different 1-year rate of RFS (stage IIIA [8% of the total number of patients]: 92.6%; stage IIID [4%]: 42.1%), and therefore AJCC-8 stage appears to be a strong prognostic factor. However, the benefit of pembrolizumab was observed across all AJCC-8 subgroups in resected high-risk stage III melanoma patients, thus suggesting that sub-staging retains no predictive importance when adjuvant therapy is administered.

## Implications for clinical practice

The new therapies introduced for stage IV melanoma do retain their efficacy also in the adjuvant setting: indeed, the best performers in stage IV are best performers in the adjuvant setting. In particular, recent developments in immunotherapy have prolonged OS in metastatic melanoma with the possibility to reach a long-term benefit [18]. Targeted therapies based on the combined BRAF and MEK inhibition also exert a long-term beneficial effect, which is more evident in patients with favorable baseline characteristics, namely normal levels of lactate dehydrogenase, with no brain metastases, and low tumor burden while elevated LDH, high tumor burden and brain metastasis should be considered as negative predictive factors [18]. This long-term benefit of targeted therapies may be related to an immune modulation: indeed, BRAF and MEK inhibitors affect tumor microenvironment and immune surveillance, and patients with complete response to targeted treatment have a pre-existing favorable immunologic signature [18].

What can we learn from the recent trials on surgery for stage III melanoma and trials in the adjuvant setting? Perhaps, the most relevant finding with immediate relevance to clinical practice comes from the Combi-AD update, presented by Georgina Long at the latest ESMO meeting. From these results it clearly stems that, at least for target therapy, adjuvant treatment prevents mainly loco-regional recurrence (− 11% with respect to placebo). Therefore, although in other recent adjuvant studies the enrolled patients underwent to an elective lymph nodes dissection before treatment, we believe that surgery could be postponed in case of loco-regional recurrence, and not performed immediately after sentinel node biopsy. This suggestion is supported by the results of the MSLT-II trial, which showed no impact of lymphadenectomy on melanoma-specific survival [4]. Of course, this applies only in case of micrometastases, as discovered by histology; in case of clinical overt recurrence, elective lymph node dissection should still be considered the first option. Moreover, patients eligible to adjuvant treatment in real life are markedly different from those included in clinical trials, as they are classified according to the new AJCC 8th classification instead of AJCC 7th classification, and also because the majority of patients in clinical practice would not have undergone CLND.

**Fig. 2:**
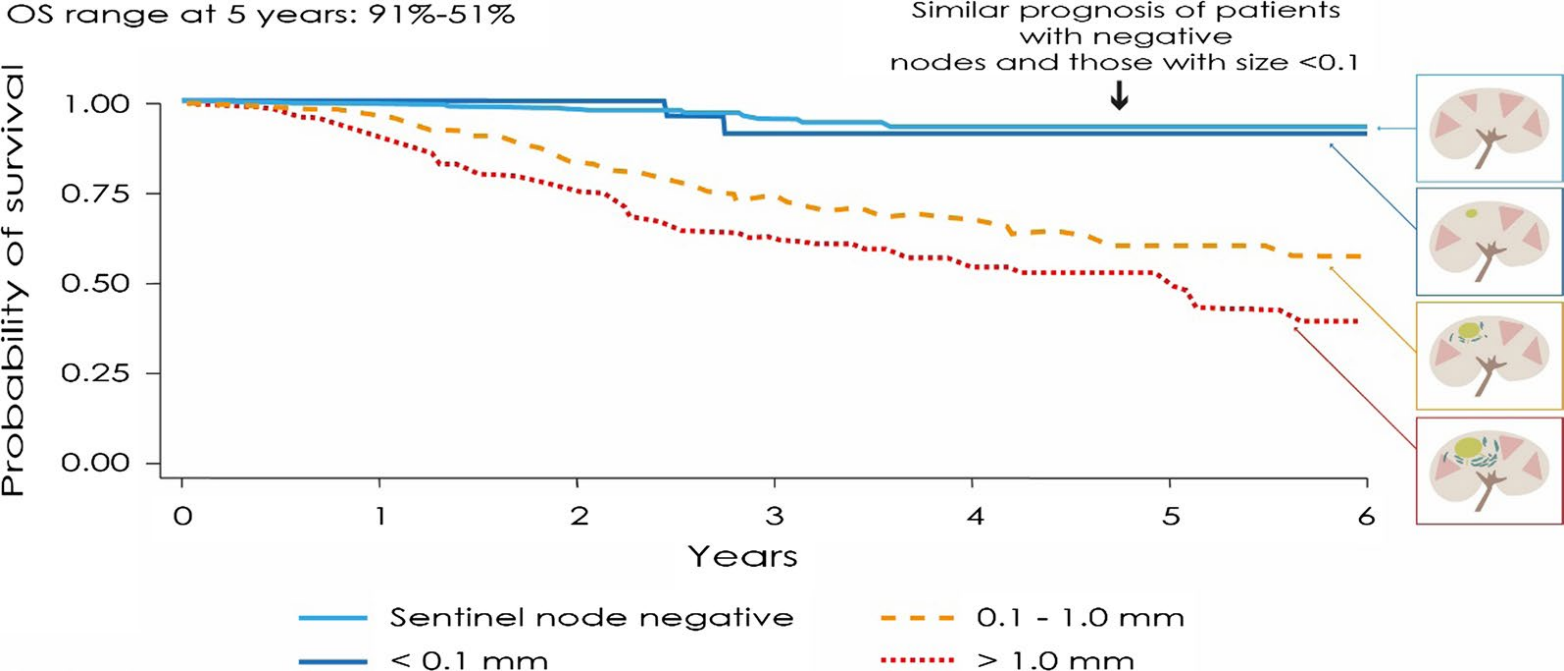
Overall survival in association with different sizes of sentinel lymph node according to Van Akkoi et al. [20]. In their study, conducted in 388 patients at three major centers within the European Organization of Research and Treatment of Cancer (EORTC) Melanoma Group, estimated overall survival at 5 years was 91% for metastasis < 0.1 mm, 61% for 0.1–1.0 mm, and 51% for > 1.0 mm (p < 0.001)

While for stage IIIB, C and D is well recognized the role of adjuvant, the role of this strategy in stage IIIA is more debated. Indeed, according to recent evidence we feel that in these patients the tumor burden within metastatic sentinel nodes should drive the decision on addressing or not them to the adjuvant treatment. In particular, a tumor burden of the sentinel nodes metastases > 1 mm allows to identify a high-risk patient, eligible to adjuvant treatment, as first suggested by van Akkooiin a landmark paper published in 2008 (Fig. 2) [10, 19]. On the other hand, in case of IIIA disease with a sentinel node metastasis < 1 mm, adjuvant should not be considered, since the risk of side effects—which can be also permanent in case of immunotherapy—is too high given the ultimate expected prognosis. Remarkably, most trials on stage IIIA enroll patients with sentinel nodes metastases > 1 mm. Overall, we suggest that T1a/T1b/T2a and N1a/N2a can be considered as selection criteria for adjuvant treatment. Moreover, more extended investigation on mutational analysis at stage III (e.g., by next-generation sequencing [20, 21]) will likely pave the way to new classification approaches.

With respect to the selection of the adjuvant therapy, at present there are no data that to indicate if it is better to use target therapy or immunotherapy as first option in the adjuvant setting (although targeted therapies can be used in patients with positive *BRAF V600* mutation). Ongoing trials will likely provide a response about this issue.

Last, preliminary evidence had suggested that checkpoint inhibitors in the neoadjuvant setting (i.e., patients with surgically palpable and resectable lymph nodes metastasis) may be superior over adjuvant therapy [22–25]. In a pilot study, 20 patients with palpable stage III melanoma were randomly assigned to ipilimumab and nivolumab, as either four courses after surgery (adjuvant arm) or two courses before surgery and two courses postsurgery (neoadjuvant arm) [21]. Neoadjuvant therapy was feasible: all patients underwent surgery at the preplanned time point, and pathological response was achieved in 7/9 patients. No relapses were reported at the time of analysis; however, 9/10 patients experienced 3/4 adverse events. In another study, 8/27 patients on neoadjuvant therapy experiences a complete or major pathological response after a single dose of anti-PD-1 therapy, and all of them remained disease-free [23]. In another randomized, phase II study treatment with combined ipilimumab and nivolumab was associated with high response rates (overall response rate: 73%, pathological complete response:, 45%) but substantial toxicity (73% grade III adverse events), while nivolumab monotherapy led to modest responses (ORR 25%, pathological complete response: 25%) and low toxicity (8% grade 3 treatment-related adverse events) [24]. Last, a randomized phase II trial in 21 patients showed that neoadjuvant plus adjuvant dabrafenib and trametinib prolonged event-free survival versus standard of care (19.7 months vs 2.9 months) in patients with high-risk, surgically resectable, clinical stage III–IV melanoma [25]. Neoadjuvant therapy appears to be feasible, although associated with a high burden of toxicity and future studies should clarify when this strategy should be preferred over adjuvant treatment [26].

## Data Availability

Not applicable.

## Abbreviations

AJCC: American Joint Committee on Cancer
CLND: completion lymph node dissections
EORTC: European Organisation for Research and Treatment of Cancer
OS: overall survival
HR: hazard ratio
RFS: relapse-free survival
ASCO: American Society of Clinical Oncology
