# Correction to: New paradigm for stage III melanoma: from surgery to adjuvant treatment

**DOI:** 10.1186/s12967-019-2067-0

**Published:** 2019-09-18

**Authors:** Paolo Antonio Ascierto, Lorenzo Borgognoni, Gerardo Botti, Michele Guida, Paolo Marchetti, Simone Mocellin, Paolo Muto, Giuseppe Palmieri, Roberto Patuzzo, Pietro Quaglino, Ignazio Stanganelli, Corrado Caracò

**Affiliations:** 1 Unit Melanoma, Cancer Immunotherapy and Innovative Therapies, Istituto Nazionale Tumori IRCCS Fondazione “G. Pascale”, Naples, Italy; 2 Ospedale Santa Maria Annunziata and University of Florence, Florence, Italy; 3 Istituto Nazionale Tumori IRCCS Fondazione “G. Pascale”, Naples, Italy; 4 Unit Melanoma and Rare Tumors, IRCCS Istituto Tumori Giovanni Paolo II, Bari, Italy; 5 Oncologia Medica B Policlinico Umberto I di Roma, Rome, Italy; 6 Surgical Oncology Unit, IOV-IRCCS of Padova and Dept. Surgery Oncology Gastroenterology, University of Padova, Padua, Italy; 7 Unit of Cancer Genetics, ICB-CNR, Sassari, Italy; 8 Research Director CNR, Italian Melanoma Intergroup (IMI), Unit of Cancer Genetics, Head Institute of Biomolecular Chemistry (ICB), National Research Council (CNR), Sassari, Italy; 9 IRCCS Fondazione Istituto Nazionale dei Tumori di Milano, Milan, Italy; 10 Dermatologic Clinic, Department of Medical Sciences, University of Turin Medical School, Turin, Italy; 11 Skin Cancer Unit, Istituto Scientifico Romagnolo per lo Studio e la Cura dei Tumori (IRST), IRCCS, Meldola, FC Italy; 12 University of Parma, Parma, Italy

## Correction to: J Transl Med (2019) 17:266 10.1186/s12967-019-2012-2

Following publication of the original article [1], the authors reported that one of the authors, Corrado Caracò, has been accidentally omitted from the author list. In this Correction the author has been added to the author list.

The correction has been implemented in the original publication of this article as well and the ‘Authors’ contributions’ section has been updated accordingly there.
